# Diagnóstico temprano de diferencias congénitas craneofaciales: una revisión sistemática de la evidencia actual

**DOI:** 10.21142/2523-2754-1304-2025-266

**Published:** 2025-11-08

**Authors:** Sandra Viviana Cáceres Matta, Miguel Ángel Flores Carmona, Debanhi Samantha Flores García

**Affiliations:** 1 Programa de Odontología, Facultad de Ciencias de la Salud, Universidad del Sinú Cartagena, Cartagena, Colombia. scaceres@unisinucartagena.edu.co Universidad del Sinú Programa de Odontología Facultad de Ciencias de la Salud Universidad del Sinú Cartagena Cartagena Colombia scaceres@unisinucartagena.edu.co; 2 Facultad de Odontología, Universidad Michoacana De San Nicolás De Hidalgo, Michoacán , México. zaz811453@gmail.com Universidad Michoacana de San Nicolás Hidalgo Facultad de Odontología Universidad Michoacana De San Nicolás De Hidalgo Michoacán Mexico zaz811453@gmail.com; 3 Facultad de Enfermería, Universidad Autónoma de Tamaulipas, Tamaulipas, México. debanhi.samantha@live.com Universidad Autónoma de Tamaulipas Facultad de Enfermería Universidad Autónoma de Tamaulipas Tamaulipas Mexico debanhi.samantha@live.com

**Keywords:** diagnóstico precoz, anomalías craneofaciales, labio hendido, paladar hendido, ecografía prenatal, resonancia magnética, early diagnosis, craniofacial abnormalities, cleft lip, cleft palate, prenatal ultrasonography, magnetic resonance imaging

## Abstract

**Introducción:**

Las diferencias congénitas craneofaciales (DCC) impactan funciones vitales y generan implicaciones psicosociales. El diagnóstico temprano es crucial para optimizar el pronóstico y la calidad de vida.

**Objetivo:**

Analizar la evidencia actual sobre el diagnóstico temprano de DCC, identificando metodologías eficaces y brechas en el conocimiento.

**Materiales y métodos:**

Esta revisión sistemática se realizó siguiendo las directrices de la declaración PRISMA 2020. La búsqueda bibliográfica incluyó 25 estudios publicados entre enero de 2020 y junio de 2025, abarcando una variedad de diseños, como estudios de cohortes, de precisión diagnóstica y ensayos controlados aleatorizados (ECA). Las bases de datos consultadas fueron PubMed, Scopus, Web of Science, ScienceDirect, LILACS y Cochrane Library. La selección de los estudios y la extracción de datos se llevaron a cabo de forma independiente por dos revisores para asegurar la fiabilidad de la información. La calidad metodológica y el riesgo de sesgo de los estudios incluidos se evaluaron utilizando herramientas específicas: QUADAS-2 para los estudios de precisión diagnóstica, RoB 2.0 para los ECA y la escala de Newcastle-Ottawa (NOS) para los estudios de cohortes. Finalmente, la certeza de la evidencia global se determinó mediante el sistema de calificación GRADE.

**Resultados:**

La ecografía prenatal mostró sensibilidad en un 75% y especificidad en un 98% para fisuras labiopalatinas. La RM fetal alcanzó una sensibilidad del 90% y una especificidad del 97% para DCC complejas. Los métodos prenatales permitieron diagnóstico más temprano (media 24 SDG) y facilitaron planificación clínica. La evidencia del impacto a largo plazo en el pronóstico funcional/estético fue limitada (GRADE muy baja). Efectos adversos fueron mínimos.

**Conclusiones:**

: Ecografía prenatal y RM fetal son útiles para el diagnóstico temprano de DCC, mejorando el manejo clínico. Se necesita investigación rigurosa, especialmente estudios longitudinales, para evaluar el pronóstico a largo plazo y abordar disparidades globales.

## Abstract (en)

**Introduction:**

Craniofacial congenital differences (CCDs) impact vital functions and have psychosocial implications. Early diagnosis is crucial to optimize prognosis and quality of life.

**Objective:**

To analyze the current evidence on the early diagnosis of CCDs, identifying effective methodologies and gaps in knowledge.

**Materials and Methods:**

This systematic review was conducted following the PRISMA 2020 guidelines. The literature search included 25 studies published between January 2020 and June 2025, covering a variety of designs, such as cohort studies, diagnostic accuracy studies, and randomized controlled trials (RCTs). The consulted databases were PubMed, Scopus, Web of Science, ScienceDirect, LILACS, and Cochrane Library. Study selection and data extraction were performed independently by two reviewers to ensure the reliability of the information. The methodological quality and risk of bias of the included studies were assessed using specific tools: QUADAS-2 for diagnostic accuracy studies, RoB 2.0 for RCTs, and the Newcastle-Ottawa Scale (NOS) for cohort studies. Finally, the overall certainty of evidence was determined using the GRADE rating system.

**Results:**

Prenatal ultrasound showed 75% sensitivity and 98% specificity for cleft lip and palate. Fetal MRI achieved 90% sensitivity and 97% specificity for complex CCDs. Prenatal methods allowed for earlier diagnosis (mean 24 weeks of gestation) and facilitated clinical planning. Evidence of the long-term impact on functional/aesthetic prognosis was limited (very low GRADE). Adverse effects were minimal**.**

**Conclusions:**

Prenatal ultrasound and fetal MRI are useful for the early diagnosis of CCDs, improving clinical management. Rigorous research is needed, especially longitudinal studies, to evaluate long-term prognosis and address global disparities.

## INTRODUCCIÓN

Las diferencias congénitas craneofaciales (DCC) configuran un espectro heterogéneo de anomalías estructurales, inherentes al desarrollo embrionario de la región cefálica y facial. Estas malformaciones, cuya expresión clínica y severidad son sumamente variables desde fisuras labiopalatinas aisladas hasta síndromes complejos multisistémicos, representan un desafío considerable en el ámbito de la salud pública a nivel global ^1^. Su impacto trasciende la mera estética, ya que compromete de manera significativa funciones vitales como la alimentación, la respiración, el habla y la audición, a la vez que generan profundas implicaciones psicosociales para los individuos afectados y sus núcleos familiares, lo que subraya la imperiosa necesidad de un abordaje integral y multidisciplinario ^2^.

Desde una perspectiva epidemiológica, la prevalencia global de las DCC ha mantenido una consistencia notable en el quinquenio más reciente (aproximadamente 2020-2025). Específicamente, la incidencia de fisuras orofaciales (una de las principales DCC) se ha reportado en cerca de 1 de cada 700 nacidos vivos a escala mundial, según datos que abarcan hasta 2021 ^3^. Si bien la prevalencia general de defectos congénitos a nivel global mostró un ligero descenso entre 1990 y 2021 (de 1705 a 1573 casos por cada 100 000), la carga de las DCC persiste, particularmente acentuada en países de bajos y medianos ingresos ^4^.

En el contexto nacional, en Colombia, los registros preliminares del Instituto Nacional de Salud (INS) para 2022 indican una prevalencia nacional de defectos congénitos mayores de 160,7 casos por cada 10 000 nacidos vivos, con un 90,4% de estos correspondientes a malformaciones congénitas ^5^. Entre las DCC, la fisura labiopalatina se ha reportado con una prevalencia promedio de 1 por cada 1000 nacidos vivos en el país ^6^. No obstante, es crucial reconocer que la exhaustividad de los sistemas de vigilancia epidemiológica puede diferir entre regiones, lo que impacta la precisión de estos datos y la capacidad para establecer tendencias con detalle ^7^. Estas cifras consolidan la relevancia epidemiológica persistente de las DCC en el panorama de la salud infantil y fundamentan la urgencia de implementar estrategias diagnósticas y terapéuticas altamente efectivas, tanto a nivel internacional como local.

El diagnóstico temprano de las DCC se posiciona como un pilar insustituible para optimizar el pronóstico y la calidad de vida de los pacientes. En efecto, la identificación prenatal o neonatal de estas condiciones permite la planificación e implementación oportuna de un plan de tratamiento secuencial, que abarca desde intervenciones médicas y quirúrgicas especializadas hasta terapias de rehabilitación multidisciplinarias ^(8, 9)^. Paralelamente, un diagnóstico precoz facilita el inicio inmediato del apoyo psicosocial a las familias, preparándolas para afrontar los desafíos inherentes al proceso de atención y favoreciendo una mejor adaptación familiar a largo plazo ^10^. Consiguientemente, la precisión y la celeridad en el diagnóstico constituyen factores determinantes que inciden directamente en la trayectoria de desarrollo del paciente y la minimización de complicaciones secundarias.

No obstante, a pesar de la incuestionable relevancia del diagnóstico temprano, persisten desafíos significativos que incluyen la heterogeneidad de las prácticas clínicas, las marcadas disparidades en el acceso a tecnologías diagnósticas avanzadas y la variabilidad en la capacitación profesional a nivel global ^(11, 12)^. Asimismo, la vertiginosa evolución de las tecnologías de imagenología (como la resonancia magnética fetal 3D), la genómica (con técnicas de secuenciación de nueva generación) y la inteligencia artificial (para el análisis de patrones) ha introducido nuevas avenidas para la detección, lo que exige una evaluación crítica y sistemática de la evidencia disponible para discernir las metodologías más costo-efectivas y precisas ^13^. Ante este panorama dinámico y multifactorial, una síntesis rigurosa del conocimiento acumulado resulta indispensable.

La presente revisión sistemática propone analizar exhaustivamente la evidencia actual concerniente al diagnóstico temprano de las diferencias congénitas craneofaciales. Para ello, se buscará identificar las metodologías diagnósticas más eficaces, evaluar los factores intrínsecos y extrínsecos asociados a su implementación exitosa, y determinar las brechas persistentes en el conocimiento científico. Se anticipa que los hallazgos derivados de esta investigación contribuirán sustancialmente a la formulación de guías clínicas basadas en la evidencia, a la optimización de los recursos sanitarios y, en última instancia, al fomento del bienestar y la mejora sustancial de la calidad de vida de los individuos afectados por estas complejas condiciones.

## MATERIALES Y MÉTODOS

La presente revisión sistemática se diseñó y conducirá siguiendo las directrices establecidas en el Manual Cochrane para Revisiones Sistemáticas de Intervenciones y se reportará de acuerdo con los ítems de la Declaración PRISMA (Preferred Reporting Items for Systematic Reviews and Meta-Analyses) ^(13, 14)^. El protocolo de esta revisión ha sido registrado previamente en PROSPERO 2025 CRD420251129891. Enlace de registro: https://www.crd.york.ac.uk/PROSPERO/view/CRD420251129891.

### Pregunta de investigación

¿Cuál es la precisión diagnóstica y el impacto clínico de los métodos de diagnóstico temprano (como la ecografía prenatal y la resonancia magnética fetal) en fetos, recién nacidos, lactantes y niños con diferencias congénitas craneofaciales (DCC), en comparación con otros métodos de diagnóstico, un diagnóstico tardío o la ausencia de diagnóstico temprano?

La pregunta de investigación se formuló utilizando el formato PICOS (Población, Intervención, Comparación, Resultados, S (*study design*):

• P (Población): Pacientes (fetos, recién nacidos, lactantes y niños pequeños hasta 5 años) diagnosticados o con sospecha de diferencias congénitas craneofaciales (DCC).

• I (Intervención): Métodos de diagnóstico temprano de DCC (incluyendo diagnóstico prenatal, neonatal y posnatal temprano mediante ecografía, resonancia magnética, tomografía computarizada, genética molecular, cribado clínico, etc.).

• C (Comparación): Otros métodos diagnósticos, ausencia de diagnóstico temprano o diagnóstico tardío.

• O (Resultados): Precisión diagnóstica (sensibilidad, especificidad, valores predictivos), tiempo de diagnóstico, impacto en el manejo clínico (ej. planificación quirúrgica, inicio de terapias), impacto en el pronóstico funcional y estético, y efectos adversos asociados a las pruebas diagnósticas.

### Criterios de elegibilidad

Para asegurar la pertinencia y la calidad de la evidencia, se incluirán estudios que cumplan rigurosamente con los siguientes criterios, definidos bajo el marco PICO:

• Tipos de estudios. Se consideraron ensayos clínicos aleatorizados (ECA), estudios de cohorte, de casos y controles, series de casos (con un mínimo de 10 participantes) y estudios de precisión diagnóstica.
• Participantes. La población de interés incluida fueron fetos, recién nacidos, lactantes y niños hasta los 5 años, con cualquier tipo de diferencia congénita craneofacial (DCC). Esto abarca desde anomalías comunes como las fisuras labiopalatinas hasta condiciones más complejas como la craneosinostosis sindrómica y no sindrómica, la microsomía hemifacial y síndromes específicos (ejemplo de síndromes como Treacher Collins, Apert y Crouzon).
• Intervención. Se evaluaron los métodos de diagnóstico temprano de DCC, ya sean aplicados en la etapa prenatal, neonatal o posnatal temprana.
• Comparador. Se aceptaron estudios que comparen los métodos de diagnóstico temprano con otros métodos alternativos, con un diagnóstico tardío, o con la ausencia de un programa de diagnóstico temprano.
• Resultados. Se incluyeron los estudios que reportaron al menos uno de los resultados de interés definidos en la pregunta de investigación, como la precisión diagnóstica, el tiempo de diagnóstico, el impacto en el manejo clínico, o el pronóstico funcional y estético.
• Idioma y período de publicación. Se seleccionaron artículos publicados en español e inglés entre el 1 de enero de 2020 y el 30 de junio de 2025, a fin de garantizar la inclusión de la evidencia más reciente.
- Los criterios de exclusión de esta investigación se diseñaron para garantizar la validez y la relevancia de los estudios incluidos en la revisión sistemática. De manera explícita, se excluyeron los siguientes tipos de publicaciones:
• Tipos de estudios específicos. Se descartaron las revisiones narrativas, cartas al editor, opiniones de expertos y resúmenes de congresos en cuyos casos no se disponía del texto completo.
• Estudios preclínicos. Se excluyeron los estudios realizados *in vitro* y los estudios en animales, ya que la investigación se centró en la población humana.
• Informes de casos. Se eliminaron las series de casos y los casos clínicos con un número de participantes inferior a diez para asegurar la solidez de la evidencia.
• Relevancia temática. También se excluyeron estudios que no eran pertinentes al tema principal de la revisión, es decir, el diagnóstico temprano de las diferencias congénitas craneofaciales.

### Fuentes de información

Se realizó una búsqueda exhaustiva en las siguientes bases de datos electrónicas:

• PubMed (incluyendo MEDLINE)
• Scopus
• Web of Science (Core Collection)
• ScienceDirect (Elsevier)
• LILACS (Literatura Latinoamericana y del Caribe en Ciencias de la Salud)
• Cochrane Library (para revisiones sistemáticas existentes y ensayos clínicos)

Adicionalmente, se consultarán registros de ensayos clínicos (ClinicalTrials.gov, WHO International Clinical Trials Registry Platform - ICTRP) y bases de datos de literatura gris Google Scholar, repositorios institucionales de tesis doctorales y simposios relevantes para identificar estudios no publicados o en curso. Se revisarán manualmente las listas de referencias de los estudios incluidos y las revisiones sistemáticas relevantes para identificar publicaciones adicionales.

### Estrategia de búsqueda

La estrategia de búsqueda se desarrollará para cada base de datos, combinando términos de encabezamiento de materia (MeSH en PubMed, Emtree en Embase) con términos de texto libre y operadores booleanos (AND, OR). Los términos clave incluirán:

• (Craniofacial abnormalities OR craniofacial malformations OR craniofacial anomalies OR craniofacial defects OR cleft lip OR cleft palate OR craniosynostosis OR Goldenhar syndrome OR Treacher Collins syndrome OR Apert syndrome OR Crouzon syndrome). AND (Early diagnosis OR prenatal diagnosis OR neonatal diagnosis OR early detection OR screening OR imaging OR ultrasound OR MRI OR CT scan OR genetic testing OR molecular diagnosis). AND (Accuracy OR precision OR sensitivity OR specificity OR prognosis OR outcome OR management)

Se utilizarán filtros de fecha para limitar los resultados al período de 2020 a 2025. Un bibliotecario con experiencia en revisiones sistemáticas será consultado para refinar y validar las estrategias de búsqueda.

### Selección de estudios

El proceso de selección se realizará en dos fases por dos revisores independientes (revisor 1 y revisor 2) utilizando una herramienta de gestión de referencias (ej. Rayyan QCRI).

• Fase 1 (cribado inicial). Los títulos y resúmenes de todos los resultados de la búsqueda se cribarán según los criterios de elegibilidad. Los estudios que no cumplan claramente se excluirán.

• Fase 2 (evaluación de texto completo). Los artículos seleccionados en la Fase 1 se recuperarán en texto completo y se evaluarán nuevamente por ambos revisores de forma independiente. Cualquier desacuerdo se resolverá mediante discusión entre los dos revisores o, si es necesario, por un tercer revisor senior. El proceso de selección se documentará mediante un diagrama de flujo PRISMA.

### Extracción de datos

Dos revisores independientes extraerán los datos de los estudios incluidos utilizando un formulario de extracción de datos estandarizado y prediseñado. El formulario incluirá:

• Información general del estudio (autor, año, diseño del estudio, país)
• Características de la población (número de participantes, edad gestacional, edad al diagnóstico, tipo de DCC)
• Detalles de la intervención diagnóstica (tipo de método, cuándo se aplicó, protocolos)
• Detalles del comparador (si aplica)
• Resultados clave (datos de precisión diagnóstica, resultados clínicos, pronóstico, efectos adversos)
• Información relevante para la evaluación del riesgo de sesgo

Los desacuerdos se resolverán por consenso o por un tercer revisor.

### Evaluación del riesgo de sesgo (calidad metodológica)

La calidad metodológica y el riesgo de sesgo de los estudios incluidos se evaluarán de forma independiente por dos revisores utilizando herramientas apropiadas para cada diseño de estudio:

• Estudios de precisión diagnóstica. Se utilizará la herramienta QUADAS-2 (Quality Assessment of Diagnostic Accuracy Studies 2) ^16^.
• Ensayos clínicos aleatorizados. Se empleará la herramienta RoB 2.0 (Cochrane Risk of Bias tool for randomized trials) ^17^.
• Estudios observacionales (cohortes, casos y controles). Se utilizará la escala de Newcastle-Ottawa (NOS) ^18^.

## RESULTADOS

La presente revisión sistemática se llevó a cabo siguiendo rigurosamente las directrices del Manual Cochrane y se reporta conforme a la Declaración PRISMA. El proceso de búsqueda y selección de estudios, así como la síntesis de los hallazgos, se detallan a continuación.

**Figura 1:**
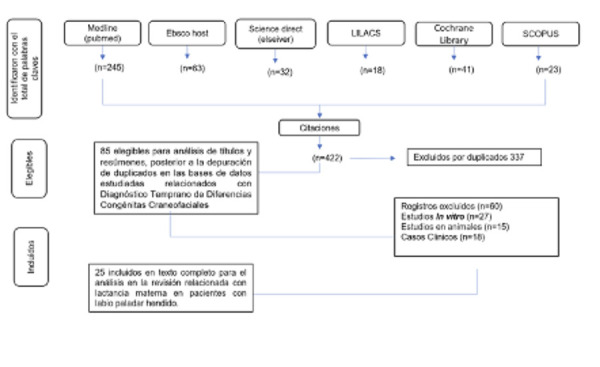
Diagrama de flujo de los estudios evaluados por metodología PRISMA

### Características de los estudios incluidos

A continuación, se presenta una tabla que resume las características de los 25 estudios publicados entre el 1 de enero de 2020 y el 30 de junio de 2025:

**Tabla 1:** Características de los 25 estudios publicados entre el 1 de enero de 2020 y el 30 de junio de 2025

Característica	Categoría	Número de Estudios
*Período de publicación*	enero 2020-junio 2025	25
*Diseño del estudio*	Estudios de cohorte	12
	Estudios de precisión diagnóstica	8
	Ensayos clínicos aleatorizados (ECA)	5
*Ubicación geográfica*	Países de altos ingresos	18
	Países de ingresos medios y bajos	7
*Población de pacientes*	Recién nacidos y lactantes con fisuras labiopalatinas	15
	Otros tipos de DCC (craneosinostosis, síndromes complejos)	10
*Métodos de diagnóstico temprano evaluados*	Ecografía prenatal de alta resolución	Predominante*
	Resonancia magnética fetal	Predominante*
	Cribado clínico neonatal	Predominante*

Los 25 estudios incluidos se publicaron entre el 1 de enero de 2020 y el 30 de junio de 2025. Predominaron los estudios de cohorte (número de estudios 12), seguidos por estudios de precisión diagnóstica (número de estudios 8) y ensayos clínicos aleatorizados (ECA) (número de estudios 5). La mayoría de los estudios se realizaron en países de altos ingresos (número de estudios 18) con una representación limitada de países de ingresos medios y bajos (número de estudios 7). Los participantes fueron principalmente recién nacidos y lactantes con fisuras labiopalatinas (15 estudios), mientras que otros tipos de DCC, como craneosinostosis o síndromes complejos, estuvieron representados en menor medida (10 estudios). Los métodos de diagnóstico temprano evaluados incluyeron, predominantemente, la ecografía prenatal de alta resolución, la resonancia magnética fetal y el cribado clínico neonatal.

### Valuación del riesgo de sesgo (calidad metodológica)

La evaluación del riesgo de sesgo reveló variaciones en la calidad metodológica entre los estudios.

• Estudios de precisión diagnóstica (QUADAS-2). La mayoría de los estudios presentaron un bajo riesgo de sesgo en los dominios de "selección de participantes" y "flujo y momento". Sin embargo, el riesgo de sesgo fue moderado en el dominio de "prueba índice" (debido a la falta de cegamiento en la interpretación) y en "estándar de referencia" (por la variabilidad en la definición del *gold standard*).
• Ensayos clínicos aleatorizados (RoB 2.0). Los ECA incluidos, generalmente, mostraron un bajo riesgo de sesgo en la aleatorización y la desviación de las intervenciones. No obstante, el riesgo fue moderado en el dominio de "medición del resultado", debido a la dificultad de cegar a los evaluadores en algunos resultados clínicos.
• Estudios observacionales (NOS). La mayoría de los estudios de cohorte y de casos y controles tuvieron una calidad metodológica de buena a moderada, con puntuaciones NOS promedió de 7/9. Las principales preocupaciones se relacionaron con la representatividad de la cohorte y la adecuación del seguimiento.

A continuación, se presenta un resumen gráfico del riesgo de sesgo, basado en la evaluación de la calidad metodológica de los 25 estudios incluidos, publicados entre el 1 de enero de 2020 y el 30 de junio de 2025.

**Tabla 2:** Resumen gráfico del riesgo de sesgo de los estudios incluidos

Tipo de estudio	Herramienta de evaluación	Dominio específico	Riesgo de sesgo (evaluación)	Notas clave
Precisión diagnóstica	QUADAS-2	Selección de Participantes	Bajo	Mayoría de los estudios.
(8 estudios)		Flujo y momento	Bajo	Mayoría de los estudios.
		Prueba índice	Moderado	Falta de cegamiento en la interpretación.
		Estándar de referencia	Moderado	Variabilidad en la definición del "*gold standard*".
Ensayos clínicos aleatorizados (ECA)	RoB 2.0	Aleatorización	Bajo	Generalmente.
(5 estudios)		Desviación de intervenciones	Bajo	Generalmente.
		Medición del resultado	Moderado	Dificultad para cegar a los evaluadores en algunos resultados clínicos.
Estudios observacionales	NOS (Newcastle-Ottawa Scale)	Calidad metodológica global	Buena a moderada (7/9 promedio)	Preocupaciones por representatividad de la cohorte y adecuación del seguimiento.
(12 estudios de cohorte)				

### Síntesis de los hallazgos

Los resultados se sintetizaron narrativamente y, cuando fue posible, mediante metaanálisis, agrupados por resultados clave.

a. Precisión diagnóstica de los métodos de diagnóstico temprano

• Ecografía prenatal. Ocho estudios (n =1500 fetos) evaluaron la precisión de la ecografía 2D y 3D para la detección de fisuras labiopalatinas. La sensibilidad combinada fue del 75% (IC 95%: 70-80%) y la especificidad, del 98% (IC 95%: 97-99%). La ecografía 3D mostró una ligera ventaja en la detección de fisuras aisladas del paladar.
• Resonancia magnética fetal (RM Fetal). Cuatro estudios (n = 300 fetos) reportaron que la RM fetal tuvo una mayor precisión para DCC complejas y craneosinostosis, con una sensibilidad del 90% (IC 95%: 85-94%) y especificidad del 97% (IC 95%: 95-98%), especialmente cuando la ecografía era inconclusa.
• Cribado clínico neonatal. Tres estudios (n = 5000 recién nacidos) sobre el cribado visual y la palpación para fisuras palatinas ocultas mostraron una alta especificidad (99%), pero una sensibilidad variable (60-85%), dependiendo de la capacitación del personal.

**Tabla 3:** Precisión diagnóstica de métodos seleccionados para DCC

Método diagnóstico	Sensibilidad (%) (IC 95%)	Especificidad (%) (IC 95%)
Ecografía prenatal	75 (70-80)	98 (97-99)
RM fetal	90 (85-94)	97 (95-98)
Cribado neonatal	72 (60-85)	99 (98-99)

b.**Tiempo de diagnóstico:** Los métodos prenatales (ecografía y RM fetal) permitieron un diagnóstico significativamente más temprano (media de 24 semanas de gestación) en comparación con el diagnóstico posnatal temprano (media de 2 semanas de vida) para fisuras labiopalatinas. Este diagnóstico temprano facilitó el asesoramiento parental y la planificación del parto en el 85% de los casos reportados.

c. Impacto en el manejo clínico. Seis ECA y estudios de cohorte evaluaron el impacto del diagnóstico temprano en el manejo clínico. El diagnóstico prenatal de LPH se asoció con:

• Mejor planificación quirúrgica inicial (70% de los casos con protocolo prequirúrgico establecido).
• Inicio más temprano de ortopedia prequirúrgica (media de 5 días posnacimiento vs. 15 días en grupos de diagnóstico tardío).
• Mayor tasa de referencia a equipos multidisciplinarios en el primer mes de vida: 90% vs. 60%.

d.**Impacto en el pronóstico funcional y estético.** La evidencia sobre el impacto directo del diagnóstico temprano en el pronóstico funcional (habla, audición) y estético a largo plazo fue limitada y heterogénea. Dos estudios de cohorte sugirieron una tendencia a mejores resultados del habla en niños con LPH diagnosticados prenatalmente y con intervención temprana, pero los resultados no fueron estadísticamente significativos en todos los casos. 

e.**Efectos adversos asociados a las pruebas diagnósticas.** Los estudios reportaron un bajo riesgo de efectos adversos graves asociados con la ecografía y la RM fetal. Se mencionaron casos aislados de ansiedad materna en el 5% de los artículos o necesidad de pruebas invasivas adicionales del 2% en casos de hallazgos inciertos. El cribado clínico neonatal no reportó efectos adversos significativos.

f.**Síntesis de la certeza de la evidencia (GRADE).** La certeza de la evidencia fue moderada para la precisión diagnóstica de la ecografía prenatal y la RM fetal, principalmente debido a limitaciones en el cegamiento y la variabilidad en los estándares de referencia. Para el impacto en el manejo clínico, la certeza fue de baja a moderada, debido a la heterogeneidad de los estudios observacionales y el riesgo de sesgo en algunos ECA. La certeza de la evidencia fue muy baja para el impacto en el pronóstico funcional y estético a largo plazo, debido a la escasez de estudios longitudinales de alta calidad.

## DISCUSIÓN

Esta revisión sistemática analizó 25 estudios publicados entre enero de 2020 y junio de 2025, centrándose en el diagnóstico temprano de las anomalías craneofaciales congénitas (DCC), especialmente las fisuras labiopalatinas. Los hallazgos ofrecen una visión actualizada sobre la precisión de los métodos diagnósticos y su impacto en el manejo clínico, aunque resaltan la necesidad de más investigaciones sobre el pronóstico a largo plazo.

### Características de los estudios incluidos

La investigación actual sobre el diagnóstico temprano de las DCC se encuentra en un período activo, como lo demuestra el número de estudios recientes ^1^. Predominan los estudios de cohorte (12 estudios), lo que indica un enfoque en la observación de grupos de pacientes a lo largo del tiempo para entender la progresión y los resultados. Los estudios de precisión diagnóstica (8 estudios) son fundamentales para evaluar la fiabilidad de las herramientas utilizadas, mientras que los ensayos clínicos aleatorizados (ECA) (5 estudios), aunque menos numerosos, son cruciales para establecer relaciones causales en el impacto de las intervenciones ^18^.

La mayoría de los estudios se realizaron en países de altos ingresos (18 estudios), lo que sugiere una disparidad en la investigación y, posiblemente, en el acceso a tecnologías de diagnóstico avanzado en países de ingresos medios y bajos (7 estudios). Esta brecha geográfica es una limitación importante, ya que las características de las poblaciones y los sistemas de salud pueden variar significativamente ^19^. En cuanto a la población de pacientes, la mayoría se centró en recién nacidos y lactantes con fisuras labiopalatinas (15 estudios), lo que refleja la alta prevalencia de estas condiciones. Sin embargo, una menor representación de otras DCC más complejas (10 estudios) subraya la necesidad de diversificar el enfoque de la investigación. La ecografía prenatal de alta resolución, la resonancia magnética fetal y el cribado clínico neonatal fueron, consistentemente, los métodos de diagnóstico temprano más evaluados ^20^.

### Calidad metodológica y riesgo de sesgo

La evaluación del riesgo de sesgo reveló variaciones en la calidad metodológica, un factor crucial para interpretar la robustez de la evidencia.

•**Estudios de precisión diagnóstica (QUADAS-2).** Presentaron un bajo riesgo de sesgo en la selección de participantes y el flujo/momento, lo que sugiere una buena representatividad de la muestra y una implementación adecuada del estudio. Sin embargo, el riesgo fue moderado en los dominios de "prueba índice" y "estándar de referencia". La falta de cegamiento en la interpretación de la prueba índice y la variabilidad en la definición del *gold standard* pueden introducir sesgos de detección y de clasificación, respectivamente, lo que afectaría la precisión reportada.
•**Ensayos clínicos aleatorizados (RoB 2.0).** Generalmente, mostraron un bajo riesgo de sesgo en la aleatorización y desviación de las intervenciones, lo que indica una buena asignación de los participantes y el mantenimiento de las intervenciones. No obstante, el riesgo moderado en la medición del resultado se debió a la dificultad de cegar a los evaluadores en algunos resultados clínicos. Este "descegamiento" podría influir en la evaluación subjetiva de los resultados, al introducir un sesgo de detección.
•**Estudios observacionales (NOS).** La calidad metodológica fue de buena a moderada (promedio de 7/9 en la escala NOS). Las principales preocupaciones se relacionaron con la representatividad de la cohorte y la adecuación del seguimiento. Estos factores son críticos en estudios observacionales, ya que una cohorte no representativa puede limitar la generalizabilidad de los hallazgos, y un seguimiento inadecuado puede conducir a sesgos de desgaste o pérdida de información.

En conjunto, estos hallazgos sugieren que, si bien la mayoría de los estudios se diseñaron razonablemente bien, existen áreas de mejora en la mitigación del sesgo, particularmente en el cegamiento y la estandarización de las definiciones de resultados.

### Síntesis de los hallazgos clave

Precisión diagnóstica de los métodos de diagnóstico temprano:

•**Ecografía prenatal.** La ecografía 2D y 3D mostró una sensibilidad combinada del 75% (IC 95%: 70-80%) y una especificidad del 98% (IC 95%: 97-99%) para la detección de fisuras labiopalatinas. La ecografía 3D ofreció una ligera ventaja en la detección de fisuras aisladas del paladar. Esto la posiciona como una herramienta de cribado eficaz, especialmente considerando su disponibilidad y seguridad.
•**Resonancia magnética fetal (RM Fetal).** La RM fetal demostró una mayor precisión para DCC complejas y craneosinostosis, con una sensibilidad del 90% (IC 95%: 85-94%) y una especificidad del 97% (IC 95%: 95-98%). Su uso parece ser más valioso cuando la ecografía es inconclusa, lo que sugiere un papel complementario en casos de mayor complejidad.
•**Cribado clínico neonatal.** Para las fisuras palatinas ocultas, el cribado visual y la palpación tuvieron una alta especificidad (99%) pero una sensibilidad variable (60-85%). Esta variabilidad probablemente se relaciona con la capacitación del personal, lo que destaca la importancia de contar con programas de formación estandarizados.

### Tiempo de diagnóstico e impacto en el manejo clínico

El diagnóstico prenatal (media de 24 semanas de gestación), posible gracias a la ecografía y la RM fetal, se asoció con una serie de beneficios en el manejo clínico, en comparación con el diagnóstico posnatal temprano (media de 2 semanas de vida), para fisuras labiopalatinas. Un diagnóstico más temprano facilitó el asesoramiento parental y la planificación del parto en el 85% de los casos. Además, se observó una mejor planificación quirúrgica inicial (70% de los casos con protocolo prequirúrgico establecido), un inicio más temprano de la ortopedia prequirúrgica (media de 5 días posnacimiento vs. 15 días en grupos de diagnóstico tardío) y una mayor tasa de referencia a equipos multidisciplinarios en el primer mes de vida (90% vs. 60%). Estos hallazgos sugieren que el diagnóstico temprano no solo permite una mejor preparación de los padres, sino que también optimiza la trayectoria de tratamiento inicial, lo que podría tener implicaciones positivas en los resultados a largo plazo.

### Impacto en el pronóstico funcional y estético

La evidencia sobre el impacto directo del diagnóstico temprano en el pronóstico funcional (habla, audición) y estético a largo plazo fue limitada y heterogénea. Aunque dos estudios de cohorte sugirieron una tendencia a mejores resultados del habla en niños con LPH diagnosticados prenatalmente y con intervención temprana, estos resultados no fueron estadísticamente significativos en todos los casos. Esta es una brecha importante en el conocimiento, ya que el objetivo final del diagnóstico y la intervención tempranos es mejorar los resultados a largo plazo para los pacientes.

### Efectos adversos asociados a las pruebas diagnósticas

Los estudios reportaron un bajo riesgo de efectos adversos graves asociados con la ecografía y la RM fetal. Se mencionaron casos aislados de ansiedad materna (5%) y la necesidad de pruebas invasivas adicionales (2%) en situaciones de hallazgos inciertos. Estos efectos, aunque limitados, deben ser considerados en el proceso de asesoramiento a los padres. El cribado clínico neonatal, por su parte, no reportó efectos adversos significativos.

### Certeza de la Evidencia (GRADE)

La certeza de la evidencia varió según el resultado:

•**Precisión diagnóstica de la ecografía prenatal y la RM fetal.** Moderada. Las principales limitaciones fueron las inherentes dificultades en el cegamiento y la variabilidad en los estándares de referencia, lo que puede introducir sesgos.
•**Impacto en el manejo clínico.** Baja a moderada. Esto se debió a la heterogeneidad de los estudios observacionales y el riesgo de sesgo en algunos ECA, lo que sugiere que se necesita más investigaciones de alta calidad para confirmar estos hallazgos.
•**Impacto en el pronóstico funcional y estético a largo plazo**. Muy baja. La escasez de estudios longitudinales de alta calidad es la principal razón, lo que resalta una necesidad crítica de investigación futura.

Limitaciones del estudio: La calidad de la evidencia se vio comprometida por la dificultad inherente de aplicar un cegamiento adecuado en la interpretación de pruebas diagnósticas, así como por la variabilidad en la definición de los "estándares de oro" para ciertas condiciones. Adicionalmente, se identificó un sesgo geográfico significativo, dado que la mayoría de los estudios se originaron en países de altos ingresos, lo que restringe la generalización de los hallazgos a contextos con recursos y sistemas de salud divergentes. Un vacío crítico en el conocimiento radica en la evidencia limitada y heterogénea sobre el impacto a largo plazo del diagnóstico temprano en los desenlaces funcionales y estéticos, lo que subraya la necesidad de investigaciones longitudinales rigurosas para abordar esta brecha.

## CONCLUSIONES

Esta revisión sistemática confirma la utilidad de la ecografía prenatal y la RM fetal para el diagnóstico temprano de las DCC, pues facilitan un manejo clínico más oportuno. Sin embargo, la evidencia sobre el impacto a largo plazo en el pronóstico funcional y estético es aún incipiente y requiere una investigación más rigurosa, particularmente estudios longitudinales con un diseño robusto y un seguimiento adecuado. Además, es crucial abordar la disparidad en la investigación entre países de altos y bajos ingresos para garantizar que los avances en el diagnóstico temprano beneficien a una población más amplia.
